# The Promising Role of Chitosan–Poloxamer 188 Nanocrystals in Improving Diosmin Dissolution and Therapeutic Efficacy against Ferrous Sulfate-Induced Hepatic Injury in Rats

**DOI:** 10.3390/pharmaceutics13122087

**Published:** 2021-12-05

**Authors:** Neamet S. Lotfy, Thanaa M. Borg, Elham A. Mohamed

**Affiliations:** Department of Pharmaceutics, Faculty of Pharmacy, Mansoura University, Mansoura 35516, Egypt; nemaezzedin@mans.edu.eg (N.S.L.); borgth@mans.edu.eg (T.M.B.)

**Keywords:** diosmin, poloxamer 188, chitosan, nanocrystals, sonoprecipitation, ferrous-sulfate-induced liver injury, iNOS expression

## Abstract

Diosmin (DSN) exhibits poor water solubility and low bioavailability. Although nanocrystals (NCs) are successful for improving drug solubility, they may undergo crystal growth. Therefore, DSN NCs were prepared, employing sonoprecipitation utilizing different stabilizers. The optimum stabilizer was combined with chitosan (CS) as an electrostatic stabilizer. NCs based on 0.15% *w*/*v* poloxamer 188 (PLX188) as a steric stabilizer and 0.04% *w*/*v* CS were selected because they showed the smallest diameter (368.93 ± 0.47 nm) and the highest ζ-potential (+40.43 ± 0.15 mV). Mannitol (1% *w*/*v*) hindered NC enlargement on lyophilization. FT-IR negated the chemical interaction of NC components. DSC and XRD were performed to verify the crystalline state. DSN dissolution enhancement was attributed to the nanometric rod-shaped NCs, the high surface area, and the improved wettability. CS insolubility and its diffusion layer may explain controlled DSN release from CS-PLX188 NCs. CS-PLX188 NCs were more stable than PLX188 NCs, suggesting the significance of the combined electrostatic and steric stabilization strategies. The superiority of CS-PLX188 NCs was indicated by the significantly regulated biomarkers, pathological alterations, and inducible nitric oxide synthase (iNOS) expression of the hepatic tissue compared to DSN suspension and PLX188 NCs. Permeation, mucoadhesion, and cellular uptake enhancement by CS may explain this superiority.

## 1. Introduction

Approximately 70–90% of active pharmaceutical compounds encounter poor water solubility, which diminishes their bioavailability [1]. Nanocrystallization is a promising tool for enhancing the solubility of many drugs due to the high loading efficiency, the cost effectiveness, and the manufacturing simplicity, which can encourage large-scale production [2]. Additionally, they can be used in the form of capsules, pellets, tablets, and nanosuspensions [3]. Nanocrystals (NCs) are solid particles in the nanometric range surrounded by a layer of a stabilizer without a matrix material [1,4]. NCs can be prepared by either top-down or bottom-up techniques [5,6]. Top-down techniques have some disadvantages, including a long preparation time, wide size distribution, high energy input, and the possible contamination by residual metal content due to too much contact time with the homogenizer [6,7]. Meanwhile, nanoprecipitation as a bottom-up technique is simple, rapid, cost-effective, and suitable for scaling up [8]. Additionally, nanoprecipitation can provide smaller NCs [6]. However, nanoprecipitation has some shortcomings, such as lower drug loading and large amount of solvent that should be removed in the processing [9]. Thus, NC preparation through procedures that combine both bottom-up and top-down methods can be beneficial [9]. Due to the high surface energy, NCs are susceptible to Ostwald ripening and crystal growth if the stabilizer is not properly selected [10]. Stabilizers are mainly divided into steric and electrostatic [11]. 

Steric stabilizers prevents crystal growth by adsorbing onto the particle surface and acts as a barrier that hinders particle aggregation. Thermodynamic driving forces for adsorption on hydrophobic drug surfaces are absent in the case of hydrophilic polymers such as hydroxypropyl methylcellulose (HPMC) and methylcellulose, but present in amphiphilic polymers such as poly vinyl pyrrolidone (PVP) and poloxamers (PLXs) [12]. Therefore, amphiphilic polymers have been preferred as steric stabilizers when compared to hydrophilic polymers. PVP is a bulky, non-toxic, non-ionic polymer which prevents particle aggregation via steric hindrance which arises from its hydrophobic carbon chains that protrude into the solvents and interact with each other [13]. PLXs are non-ionic linear triblock copolymers formed of a central hydrophobic chain of poly propylene oxide (PPO) and two hydrophilic tails of polyethylene oxide (PEO) [1,14]. Hydrophobic PPO heads are adsorbed on NC surfaces while hydrophilic PEO chains protrude towards the aqueous environment, providing a steric shield that prevents crystal aggregation and growth [15,16]. These polymers can decrease the interfacial tension and increase NC wettability [1]. Additionally, PLXs can interact with hydrophobic surfaces and biological membranes due to their amphiphilic nature [17]. Moreover, PLX188 can act as a permeation enhancer because of P-glycoprotein and a cytochrome P3A4 inhibitor possibly affecting the pharmacokinetics of some orally administered drugs [18]. 

Electrostatic stabilization is based on the formation of repulsive Coulomb forces between the charged colloidal particles. They include anionic surfactants such as sodium dodecyl sulfate (SDS) and charged polymers such as chitosan (CS) [19]. CS is a natural polysaccharide extracted from chitin (β-1,4 linked N-acetyl glucosamine) by partial deacetylation [20]. CS is non-toxic, biodegradable, and biocompatible [21]. It exhibits high solubility at the acidic pH of the stomach, as well as mucoadhesive and permeability-enhancing properties due to the protonation of its amino groups at acidic media providing electrostatic interactions with the negatively charged gastrointestinal mucosa [22]. The positively charged CS nanoparticles can augment the interaction with the negatively charged cellular membranes; hence, they can accelerate their intracellular uptake [23]. Additionally, positively charged nanoparticles with a diameter ranging from 200 to 500 nm have been reported to passively target liver macrophages to a greater extent than neutral macrophages [24]. NCs may fail in vivo due to drug precipitation and the rapid transit time from the stomach to the intestines [25]. Therefore, NCs coated with CS could enhance drug delivery, particularly to the liver, due to its variable above-mentioned effects.

Diosmin (DSN) (3,5,7-trihydroxy-4-methoxy flavone-7-rutinoside) is a natural flavonoid glycoside derived from hesperidin by dehydrogenation [26]. It is used for the treatment of hemorrhoids, chronic venous insufficiency, lymphedema, and varicose veins [27]. It possesses diverse pharmacological activities, mainly due to its free radical scavenging ability. These include anti-inflammatory [28], anticancer [29,30], antiulcer [31], antihyperlipidemic [32], and antihyperglycemic activities [33]. Additionally, it has been reported that DSN exerted a hepatoprotective activity against iron-induced liver damage [34] and reduced the liver injury caused by carbon tetrachloride and γ-radiation [35]. However, a low dissolution rate and an impaired gastrointestinal absorption have been reported for this drug [36,37]. Orally administered DSN is rapidly hydrolyzed by intestinal microflora enzymes into its aglycone diosmetin, which is enzymatically esterified to its metabolite of 3,7-O-diglucuronide [36,37]. Therefore, a large oral dose (500 mg twice daily) is required; however, low and highly variable diosmetin concentrations have been recorded in plasma after a single oral dose of DSN [38]. Inconsistent absorption due to the rapid transit time could be diminished by mucoadhesion to the gastrointestinal wall, replenishing the absorbed drug. Nanoparticles can effectively bind to the mucus network [39]. 

The low solubilizing capacity of most lipids and surfactants towards DSN reflects its rigid nature, being insoluble in water and most organic solvents [40]. To enhance DSN solubility and delivery, it has been formulated in the form of phytosomes [38] and nanofibers [27]. Additionally, complexation with hydroxypropyl-β-cyclodextrin has been attempted [26]. In addition, DSN nanosuspensions stabilized with HPMC have been reported [41]. The latter study of DSN nanosuspensions did not involve the use of amphiphilic polymers such as PVP and PLXs, which have been recommended as more effective steric stabilizers of nanosuspensions than hydrophilic polymers due to the absence of the thermodynamic driving force of the latter to enable their adsorption onto hydrophobic drug surfaces [12]. A combination of steric and electrostatic stabilizers may provide more effective stabilization of NCs [42]. Such a combination was not attempted in this study. Moreover, there has been no investigation of the possible potentiating effects of selected NCs on DSN therapeutic efficacy. 

Therefore, the aim of this study was to improve DSN dissolution and delivery after oral administration. To fulfill this target, DSN NCs were developed, combining steric and electrostatic stabilization strategies aiming to attain enhanced NC stabilization and therapeutic efficacy. In vitro evaluation of the selected NCs was attempted. Furthermore, the effects of the selected DSN NCs on the therapeutic efficacy against ferrous sulfate-induced liver damage in rats were investigated through serum and tissue biomarker assessments, histopathological examinations, and the immunohistochemical localization of inducible nitric synthase (iNOS). 

## 2. Materials and Methods

### 2.1. Materials

Diosmin (DSN) was a kind gift from Sigma Pharmaceuticals Industries, Quesna, Egypt. Poloxamer 188 (PLX188), reduced glutathione (GSH), hepatic malondialdehyde (MDA) kits were purchased from Sigma-Aldrich Co., St Louis, MO, USA. Poly vinyl pyrrolidone K25 (PVP-K25) and sodium dodecyl sulfate (SDS) were supplied by Sedico Pharmaceutical Company, Cairo, Egypt. Low-molecular-weight chitosan (CS, M wt, 110–150 kDa, Chitoclear HQG 10, Code No:43000, Batch No. TM4870, deacetylation degree 95%) was kindly donated by Primex, Siglufjörður, Iceland. Dimethyl sulfoxide, glacial acetic acid (99%), sodium hydroxide, sodium carboxymethyl cellulose, formalin, hydrogen peroxide (H_2_O_2_), and ferrous sulfate were purchased from Adwic, EL Nasr Pharmaceutical Chemicals Co., El Khanka, Egypt. Serum alanine amino transferase (AST), aspartate aminotransferase (ALT), albumin (ALB) and total bilirubin (TB) kits were purchased from Diamond Diagnostics, Holliston MA, USA. The serum gamma glutamyl transferase (GGT) kit was merchandized from Analyticon, Lichtenfels Germany. The serum lactate dehydrogenase (LDH) kit was procured from Biosystems, Barcelona, Spain. The serum total cholesterol (TC) kit was acquired from Spinreact, Girona, Spain. The serum alkaline phosphatase (ALP) kit was obtained from Biodiagnostic, Giza, Egypt. 3,3′-diaminobenzidine tetrahydrochloride (Substrate Kit, DAB) was obtained from Vector Laboratories Inc., Burlingame, CA, USA. The tissue-inducible nitric oxide synthase (iNOS) primary antibodies were procured from Proteintech, Rosemont, IL, USA. All other chemicals were of fine analytical grades.

### 2.2. Preparation of DSN NCs

**Figure 1:**
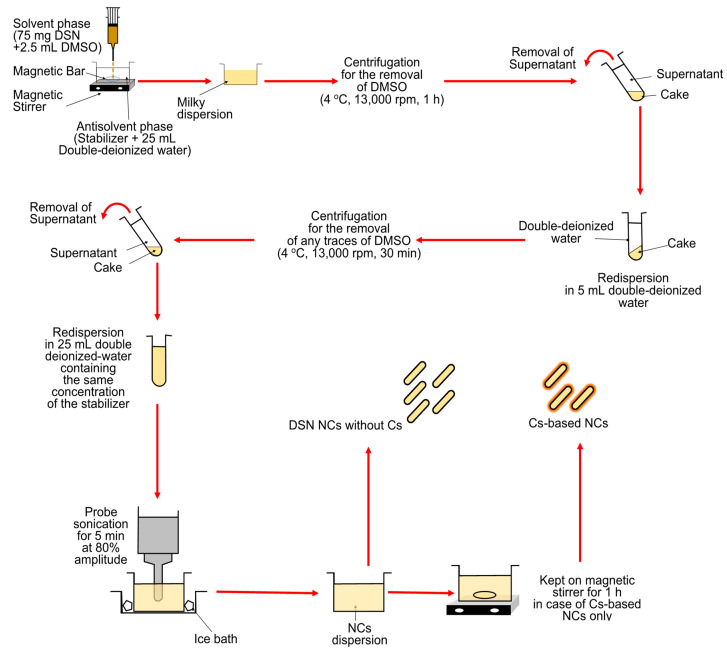
Schematic representation of the DSN NCs preparation method.

DSN nanocrystals (NCs) were prepared through procedures that combined the bottom-up technique of antisolvent precipitation and top-down method of probe sonication [43,44]. A schematic representation of the preparation method is illustrated in Figure 1. Briefly, DSN (75 mg) was dissolved in 2.5 mL dimethyl sulfoxide and added dropwise to 25 mL double-deionized water containing PVP K25, PLX188, and SDS, used individually as stabilizers at different concentrations of 0.1%, 0.15%, 0.3%, and 0.6% *w*/*v* (Table 1). The resultant dispersions were centrifuged at 13,000 rpm for 60 min at 4 °C using a cooling centrifuge (CE16-4X100RD, ACCULAB, New York, NY, USA). The obtained cake was washed with double-deionized water and recentrifuged for 30 min to remove any residues of dimethyl sulfoxide [9]. NCs were carefully collected and resuspended in the same concentration of each of the utilized stabilizers to be stabilized during storage [45]. Probe sonication has been reported to provide smaller and more homogeneous NCs due to the direct contact with the dispersion and the transfer of a high energy output rather than the energy spreading, as in the case of a bath sonicator [13]. As a preliminary study, different sonication amplitudes (50%, 80% and 100%) and times (2.5, 5 and 10 min) were tested and the smallest size reduction in NCs using the examined stabilizers at a concentration of 0.15% *w*/*v* was obtained by probe sonication at an 80% amplitude for 5 min (data not shown). Accordingly, NC dispersions were then sonicated using a probe sonicator (Serial No. 2013020605, Model CV 334) attached to a homogenizer (SONICS vibra cell^TM^, model VC550, sonic & materials Inc., Newtown, CT, USA) at 80% amplitude for 5 min and the temperature was kept constant using an ice bath [13].

CS-based NCs of DSN were prepared by employing the above-mentioned procedure with a modification. Firstly, CS was dissolved in 0.1% *v*/*v* acetic acid to obtain three solutions of different concentrations (0.1%, 0.2% and 0.3% *w*/*v*). Five milliliters of each CS solution was diluted with double-deionized water containing the selected stabilizer at its optimum concentration to a final volume of 25 mL as an aqueous phase to obtain different concentrations of CS (0.02%, 0.04% and 0.06% *w*/*v*). The produced dispersions were kept on a magnetic stirrer (MS300HS, MTOPS Corp., Korea) for 1 h.

### 2.3. Characterization of DSN NCs

#### 2.3.1. Particle Size and ζ-Potential Measurements

The average diameters of the prepared NCs were determined by dynamic light scattering technique (Zetasizer, Malvern Instruments Ltd., Malvern, Worcestershire, UK). An aliquot of the prepared samples was taken, properly diluted (1:100) with double-deionized water, and analyzed for particle size, polydispersity index (PDI) and ζ-potential. All measurements were performed in triplicate and the results are expressed as the average value ± standard deviation (SD).

#### 2.3.2. Transmission Electron Microscopy (TEM)

The morphology of the selected NCs with and without CS was examined using a transmission electron microscope (JEOL JEM-2100, JEOL Ltd., Tokyo, Japan) operating at an accelerating voltage of 160 kV. Each sample was properly diluted (1:10) and sonicated for 2 min. One drop was added on a Formvar-coated copper grid (200 meshes, Science Services, Munich, Germany). The excess material was removed with a filter paper. After complete drying at room temperature, the image was captured.

#### 2.3.3. Scanning Electron Microscopy (SEM)

The surface properties of DSN powder and the lyophilized selected NCs, either with or without CS, were examined using scanning electron microscopy (SEM) (JSM (JSM-6510 LV; JEOL, Tokyo, Japan). Samples were mounted on a metal stub using double-sided adhesive carbon tapes. They were coated with a gold layer and monitored under SEM at 30 kV.

#### 2.3.4. Solid-State Characterization 

##### Fourier-Transform Infrared (FT-IR) Spectroscopy

FT-IR spectra of DSN powder, the selected stabilizer, CS, the physical mixtures (PMs) of DSN and the selected stabilizer with or without CS, as well as the lyophilized NCs based on the stabilizer alone or combined with CS, were recorded using an FT-IR spectrophotometer (Madison Instruments, Middleton, WI, USA). The samples were homogenized with KBr to then be compressed into discs and scanned at the range of 4000–400 cm^−1^.

##### Differential Scanning Calorimetry (DSC)

The thermal behavior of the above-mentioned samples was assessed employing differential scanning calorimetry (DSC) (model DSC-PerkinElmer Inc., Waltham, MA, USA). Five milligrams of each sample was placed into an aluminum crucible and sealed using an aluminum lid by a sealing machine. Then, they were heated from 35 °C to 350 °C at a rate of 10 °C/min under constant dry nitrogen purging at 20 mL/min. Indium with a purity of 99.99% and a melting point of 156.6 °C was used to calibrate the temperature. 

##### X-ray Diffractometry (XRD)

X-ray diffractometry (XRD) patterns of the above-mentioned samples were obtained (Diano, Woburn, MA, USA) The diffraction patterns were recorded in a step-scan model at a voltage of 45 kV and a current of 9 mA in a range of 3° to 55° (2θ) with a step size of 0.020°.

### 2.4. Lyophilization of NC Dispersions

Lyophilization is recommended to attain long-term stability and easy handling. Due to the high energy, NCs can easily be subjected to crystal growth; thus, water removal is required to protect against this growth [11]. After lyophilization, rapid reconstitution of the lyophilized powder and maintaining the particle size distribution are very important to ensure high product quality [46]. However, lyophilization may affect the product quality through particle aggregation. To avoid such undesirable effects, a cryoprotectant can be added. 

Mannitol has been presented as a promising cryoprotectant because it crystallizes around the nanoparticles, creating a glassy/vitreous coat that protects them from the mechanical stress of ice crystals, thereby preventing aggregation [47]. Moreover, mannitol is one of the most commonly used matrix formers in freeze-dried pharmaceutical products because of its crystallinity, high eutectic temperature, and matrix properties [13]. It crystallizes during freezing and enables drying at higher product temperatures and with higher sublimation rates relative to purely amorphous systems. This promotes efficient freeze-drying as well as a physically stable and a pharmaceutically elegant freeze-dried product. In addition, mannitol crystallization during freezing could allow amorphous components to be dried above the critical temperature, preventing the cake collapse and reducing the drying times [13,48]. Therefore, mannitol (1% *w*/*v*) was added as a cryoprotectant to the examined PLX188 NC dispersion either with or without CS before freezing at −80 °C [6,45,49]. Lyophilization was then performed at −45 °C under 7 × 10^−2^ mbar pressure (Labconco, LYPH.LOCK 4.5, Kansas, MO, USA). The lyophilized powders were kept in a desiccator for characterization. Particle size analysis of the lyophilized powders after reconstitution with double-deionized water was carried out, and the results were compared to those of the freshly prepared nanosuspensions as well as those of the lyophilized powders of the selected nanosuspensions without the addition of mannitol.

### 2.5. Drug Content Determination

For drug content determination, the accurately weighed (0.01 g) lyophilized powder was well mixed with 10 mL 0.2 N sodium hydroxide and filtered through a 45 µm Millipore filter to remove any undissolved material such as CS. The filtrate was properly diluted and analyzed spectrophotometrically (ultraviolet/visible [UV/VIS] spectrophotometer; JASCO, Tokyo, Japan) at 268 nm against a blank of the corresponding lyophilized plain NCs that was treated the same to eliminate interference by components other than the drug, if any. The experiment was performed in triplicate and the average drug content (%) and SD were calculated.

### 2.6. In Vitro Dissolution Study

Dissolution studies were carried out using USP apparatus II (paddle method) (Dissolution Apparatus USP standards, scientific, DA-6D, Bombay, India). It has been reported that DSN exhibits pH-dependent solubility (pk_a_ = 9.39–10.12); hence, it exhibits poor solubility in an acidic medium (pH 1.2) and a neutral (pH 7.4) buffering system [41]. The authors added that DSN solubility was highly increased as the pH was shifted to the alkaline range. As a preliminary study, borate buffer pH 10 was tested as a dissolution medium and the results (not shown) indicated non-sink conditions of both DSN powder and DSN–PLX188 NCs, as revealed by a plateau of the percentage drug dissolved (about 20% and 60%, respectively) at 5 min and 1 h, respectively, followed by a gradual decline rather than an increase until 8 h. These results can be explained by the inadequate solubilization capacity of this medium; hence, the critical supersaturation ratio beyond which crystal formation occurs may have been exceeded. Drug precipitation and a decline in the proportion of drug dissolved after reaching a plateau can result from crystal growth [50]. Consequently, sodium orthophosphate buffer (pH 12) has been used due to its better solubilization capacity for DSN [41] that can provide distinct dissolution profiles of the tested NCs and DSN powder and avoid exceeding the critical supersaturation ratio and subsequent crystal growth and drug precipitation [50]. Therefore, the dissolution medium in this study consisted of 500 mL sodium orthophosphate buffer pH 12 that was stirred at 100 rpm. The temperature was maintained at 37 ± 0.5 °C. Fifty milligrams of DSN powder, or an equivalent amount of the lyophilized selected DSN NCs with and without CS, was introduced into the stirred dissolution medium. At predetermined time intervals (5, 10, 20, 30 and 45 min, as well as 1, 2, 3, 4, 5, 6, 7 and 8 h), samples (3 mL) were withdrawn and replaced with the same volume of the fresh dissolution medium to then be filtered through a 0.22 µm Millipore filter. The filtrates were properly diluted and analyzed spectrophotometrically (ultraviolet/visible [UV/VIS] spectrophotometer; JASCO, Tokyo, Japan) at 268 nm against a blank of the corresponding plain NCs that was treated the same as the examined medicated NCs to avoid interference by components other than the drug, if any. All measurements were performed in triplicates and the results are presented as the mean ± SD. The dissolution parameters including the percentage dissolved after 2 h (PD_2h_), mean dissolution time (MDT), dissolution efficiency (DE) and similarity factor (*f*_2_) were calculated based on the literature [51].

### 2.7. Kinetics Analysis of DSN Release Data

To describe the release mechanism of DSN from the selected CS-based NCs, in vitro release results were analyzed according to different kinetic models, including zero-order, first-order [52] and Higuchi models [53]. The Korsmeyer-Peppas model was applied to establish the drug release mechanism according to the following equation: M_t_/M_∞_ = k t^n^, where M_t_ represents the amount of drug released at a time t, M_∞_ is the amount of drug released in infinite time, M_t_/M_∞_ is the fraction of the drug released after time t, k is the kinetic constant, and n is the diffusional exponent which is equal to the slope of log M_t_/M_∞_ against log t [54]. The kinetic model that expressed the drug release mechanism was chosen based on the highest coefficient of determination (R^2^) value.

### 2.8. Stability Studies

Stability studies were performed using the lyophilized DSN NCs based on the selected stabilizer either alone or in combination with CS during a storage period of three months at room temperature (25 ± 2 °C). The samples were stored in screw-capped amber glass containers to be reconstituted in double-deionized water and then examined initially and monthly regarding particle size, PDI, ζ-potential, and percentage drug content, as previously discussed.

### 2.9. In Vivo Evaluation

#### 2.9.1. Animals

All animal handling and procedures were performed in compliance with the U.S. National Institute of Health Guide for the Care and Use of Laboratory Animals (NIH publication No. 85–23, revised 1996). The protocol was approved by the Ethical Committee of Faculty of Pharmacy, Mansoura University, Egypt (ethical approval code 2017-65 on 30 July 2017). Animals were kept under regular 12 h light/12 h dark cycles at a temperature of 25 ± 1 °C and a relative humidity of 55 ± 5%. Free access to a standard laboratory food and water was allowed.

Sixty male Wistar albino rats weighing 200–220 g were divided into ten groups (six rats per each group). Intragastric gavage was utilized to enable oral administration. In addition to the pretreatment regimen, after treatment, one rat was followed to assess the possibility of potentiated uptake of the examined NCs, particularly the positively charged ones incorporating CS by overexpressed Kupffer cells in the inflamed hepatic tissue [24]. Therefore, both treatment regimens were followed to examine the efficacy of DSN either as a drug suspension or selected NCs against ferrous-sulfate-induced liver injury in comparison with normal and positive control groups.

#### 2.9.2. Pretreatment Model

Sodium carboxymethylcellulose (Na CMC) at a concentration of 1% *w*/*v* was used as a suspending agent of DSN suspension to allow a uniform dose following shaking. Therefore, rats of normal and positive control groups received 1% *w*/*v* Na CMC orally for 10 consecutive days to examine its effects on the liver, if any. Oral pretreatment with 50 mg/kg DSN suspended in 1% *w*/*v* Na CMC or an equivalent amount of DSN NCs based on either the selected stabilizer alone or combined with CS was accomplished for 10 consecutive days. Rats of all groups, except normal controls, were intraperitoneally (i.p) injected with ferrous sulfate at a dose of 30 mg/kg [34] on the 9th and 10th days after the respective pretreatments. 

#### 2.9.3. Post-Treatment Model

Similarly, rats of normal and positive control groups received 1% *w*/*v* Na CMC orally for 10 consecutive days. All groups except the normal control were injected i.p with ferrous sulfate at a dose of 30 mg/kg [34] on the 1st and 2nd days. Rats of the third group were administered DSN suspended in 1% *w*/*v* Na CMC orally at a dose of 50 mg/kg for 10 consecutive days, starting from the 2nd day after the induction dose. An equivalent amount of DSN NCs based on the chosen stabilizer either alone or together with CS was orally given to the fourth and fifth groups, respectively, starting from the 2nd day after the induction dose.

#### 2.9.4. Blood and Tissue Collection

Blood and tissue samples were collected according to a method described in the literature [34]. On the 11th day in the pretreatment model and on the 12th day in the post-treatment model, rats were anaesthetized by ether and blood samples were collected from the retro-orbital plexus in centrifuge tubes using heparinized micro-capillary tubes. Rats were then sacrificed. Following animal sacrificing, the abdominal cavities were opened, and livers were carefully isolated to be washed with ice-cooled saline. The median and left lobes were separated to prepare liver homogenates for oxidative stress and inflammatory biomarker assessment, as well as tissue specimens for histopathological and immunohistochemical examinations.

#### 2.9.5. Biomarkers Assessment 

Blood samples were allowed to coagulate at room temperature to then be placed in a water bath maintained at 37 ± 0.5 °C for 10 min and centrifuged at 10,000 rpm for 20 min to separate the clear sera [34]. The sera were analyzed for biochemical markers, including alanine aminotransferase (ALT), aspartate aminotransferase (AST), albumin (ALB), total bilirubin (TB), gamma glutamyl transferase (GGT), alkaline phosphatase (ALP), total cholesterol (TC) and lactate dehydrogenase (LDH) using the respective commercial kits. 

One gram of the liver median lobe was homogenized with 5 volumes of isotonic ice-cooled normal saline for preparation of the 20% liver homogenate. The resultant homogenates were employed for the determination of oxidative stress biomarkers, including hepatic malondialdehyde (MDA) and reduced glutathione (GSH), as well as inflammatory biomarkers and nitrate/nitrite production (NO_x_). GSH content in the liver homogenates was determined according to a previously adopted method [55]. The hepatic content of MDA as thiobarbituric acid reactive species was assayed in the liver homogenates to assess the lipid peroxidation [56]. Additionally, NO_X_ production was evaluated in the liver homogenates [57].

#### 2.9.6. Histopathological Examination 

Hepatic tissue samples were fixed in 10% (*v*/*v*) buffered formalin solution for 24 h. The fixed samples were washed, dehydrated by ascending grades of ethyl alcohol, cleared in xylene, and embedded in a melted paraffin wax in a hot air oven (56 °C). Paraffin blocks were divided into two sets of sections. Each was made 5 μm thick using a microtome and picked up on a clean glass slide. At the time of staining, the first set of the paraffin sections was deparaffinized, rehydrated in descending grades of ethyl alcohol and routinely stained with hematoxylin and eosin (H&E), in accordance with a reported method [58]. The stained sections were dehydrated in ethyl alcohol, cleared in xylene, cover-slipped and examined under a light microscope (Leica Microsystems). The liver specimens were examined by an experienced pathologist in a blinded manner. Hepatic microscopic damage was scored in all groups based on previously reported criteria [59]. In terms of inflammatory cell infiltration, the specimens were differentiated into four grades: grade 0, no infiltration; grade 1, one to two foci per 200× field; grade 2, three to four foci per 200× field; and grade 3, more than four foci per 200× field. With respect to the hepatocellular degeneration, the specimens were classified into three grades: grade 0, no ballooning; grade 1, a few balloon cells; and grade 2, many cells/prominent ballooning. Hepatic fibrosis was recognized as five stages: stage 0, no fibrosis; stage 1, mild, perisinusoidal or periportal fibrosis; stage 2, moderate, perisinusoidal and periportal fibrosis; stage 3, bridging fibrosis; and stage 4, cirrhosis.

#### 2.9.7. Immunohistochemical Evaluation of Inducible Nitric Oxide Synthase (iNOS) 

iNOS is one of the three key enzymes that generate nitric oxide from the amino acid L-arginine; hence, increased iNOS activity indicates tissue damage [60]. iNOS evaluation was accomplished according to an immuno-enzymatic staining method [61]. This method is based on the binding of primary antibodies to its antigen in examined tissue sections, followed by binding the biotinylated secondary antibodies to the primary antibodies. Then, an enzyme–conjugate binds to the biotin on the secondary antibodies to be detected by a substrate–chromogen mixture with a color intensity depending on the activity of the bound enzyme. 

The second paraffin section set was used to immunohistochemically evaluate iNOS activity in the hepatic tissue. Paraffin sections were deparaffinized using xylene for 30 min and then rehydrated in descending grades of ethanol. The blockage of endogenous peroxidase activity was attained by incubation in 3% hydrogen peroxide (H_2_O_2_) for 15 min at room temperature. This was followed by boiling with 0.01 M sodium citrate buffer (pH 6) for 20 min in a microwave oven to promote immunoreactivity and counteract the antigenicity loss that might happen due to some epitopes of formalin-fixed paraffin-embedded tissues. The slides were allowed to cool and washed four times with phosphate-buffered saline (pH 7.2). Primary antibodies (dilution 1:100) were added and the tissue sections were incubated at room temperature for 60 min in a humidity chamber, which was a covered Petri dish and a filter paper moistened with water and fixed on the dish bottom to prevent the specimens drying. After that, incubation with the biotinylated secondary antibodies was allowed for 10 min and the enzyme conjugate was added to the specimens to then be incubated for 10 min. Finally, the sections were rinsed with Tris-buffered saline and the immunoreaction was visualized using 3,3′-diaminobenzidine tetrahydrochloride (DAB). Sections were washed for 10 min under running tap water, and then counterstained with Mayer’s hematoxylin. Staining intensity of the positively stained cells was evaluated using a digital camera (Olympus Corporation, Tokyo, Japan) mounted on a light microscope (Leica Microsystems, Wetzlar, Germany). The intensity of the immunohistochemical staining as an indication of the extent of iNOS activity was scored as follows: 0, negative; 1, weak; 2, moderate; and 3, strong [62]. All evaluations were blindly inspected by a pathologist.

### 2.10. Statistical Analysis

Statistical analysis using one-way ANOVA, followed by Tukey–Kramer multiple comparisons tests, was carried out employing GraphPad Prism version 5.00 (GraphPad software, San Diego, CA, USA). The results were statistically analyzed at the significance level of *p* < 0.05.

## 3. Results and Discussion

### 3.1. Particle Size and ζ-Potential Measurements

The results of particle size and PDI analyses are listed in Table 1. Generally, a uniform particle size distribution was verified by PDI ≤ 0.55 ± 0.02. The stabilizer concentration was critical. Initially, the increase in their concentrations from 0.1% *w*/*v* to 0.15% *w*/*v* resulted in smaller nanoparticles. A greater increase in PVP K25 concentration beyond 0.15% *w*/*v* produced significantly larger nanoparticles. The increase in PLX188 concentration to 0.3% *w*/*v* insignificantly affected the particles size, although a higher content (0.6% *w*/*v*) of this stabilizer significantly enlarged the particles. Regarding SDS, a further size reduction was recorded on the increase in SDS concentration to 0.3% *w*/*v*; however, significant particle enlargement was noticed beyond such a concentration (0.6% *w*/*v*). In other words, the optimum stabilizer concentration that produced the smallest particle size was 0.15% *w*/*v* for both PVP K25 and PLX188, and 0.3% *w*/*v* for SDS. These results can be explained on the basis that at a low concentration, the stabilizer would be insufficient to completely cover the NC surface and decrease the surface free energy; hence, aggregation could be expected. At the optimum stabilizer concentration, the adsorbed sterically stabilizing layer would have the sufficient thickness and provide a stable NC dispersion [11,63]. Beyond the optimum stabilizer concentration, NC size increases might be due to the Ostwald ripening phenomenon caused by a rise in drug solubility initiated by the stabilizers [64,65]. Moreover, the increase in the stabilizer concentration beyond the optimum value could impart a high medium viscosity that might hinder the transmission of ultrasonic waves and particle diffusion during antisolvent displacement, leading to NC enlargement [66].

Significantly smaller NCs were obtained with PLX188 than that recorded when PVP K25 or SDS were used at the same concentration. Among the studied stabilizers, the smallest particle diameter (350.20 ± 1.79 nm) was produced when PLX188 was used. Accordingly, PLX188 at a concentration of 0.15% *w*/*v* was selected as a steric stabilizer of DSN NCs. Hence, the ζ-potential of PLX188 NC dispersions was measured (Table 2). The results indicated negative ζ-potential values in spite of the nonionic nature of PLX188, possibly because of the polar and electronegative nature of DSN that can be attributed to the deprotonation of its phenolic –OH groups [67]. Interestingly, the greatest ζ-potential was attained at a concentration of 0.15% *w*/*v* of this stabilizer, and the potential decreased with its higher concentrations (0.3% and 0.6% *w*/*v*). These results can be explained on the basis that the ζ-potential decreased with the increase in the stabilizer layer thickness due to the outward shift of the slipping plane at which the zeta potential was measured; thus, the charge density was much smaller than that on the surface, resulting in lower recorded ζ-potential values [6,68,69]. The greatest ζ-potential of PLX188 NC dispersion based on 0.15% *w*/*v* of this stabilizer may counteract NC aggregation and enlargement, and hence account for the smallest size recorded for these NCs among the examined dispersions.

Based on the above-mentioned results, PLX188 was combined with CS as an electrostatic stabilizer aiming to provide more stable DSN NCs as well as an enhancement of DSN delivery due to the expected permeation, mucoadhesion and cellular uptake potentiation capabilities of CS. CS at a concentration of 0.02% *w*/*v* resulted in significant NC enlargement when compared to those prepared using PLX188 (0.15% *w*/*v*) alone. These results can be attributed to the partial coverage of DSN NCs by CS. However, the increase in CS concentration to 0.04% *w*/*v* significantly lowered the particle size again, possibly due to the complete coverage of the particles surface by CS and the higher electrostatic stability, as indicated by the significantly greater ζ-potential values compared to the 0.02% *w*/*v* CS-based NC dispersion that could strongly counteract NC aggregation, and hence, the particle enlargement. Accordingly, a rise in CS content to 0.06% *w*/*v* insignificantly affected ζ-potential, possibly due to the complete coverage of the particle surface that was previously accomplished when this polymer was used at 0.04% *w*/*v*. However, such increments in the CS concentration imparted significantly larger NCs. The subsequent size increase can be explained by multilayer CS accumulation on the particle surface [37]. Successful coating of DSN NCs with CS was reflected by the positive ζ-potential due to CS protonated amine groups that masked the original negative charge of DSN hydroxyl groups. 

In light of the results of particle size and ζ-potential analysis, the selected DSN NCs dispersion to be further investigated was that based on 0.15% *w*/*v* PLX188 combined with 0.04% CS, because CS incorporation did not significantly affect NCs size, although increased their ζ-potential when compared to PLX188 NCs. The resultant positive ζ-potential might augment the interaction with the negatively charged cellular membranes; hence, their intracellular uptake [23]. Moreover, positively charged nanoparticles with diameters ranging from 200 to 500 nm have been reported to passively target liver macrophages to a greater extent than neutral nanoparticles [24]. These results indicate the benefits of our study in comparison with a previous study of DSN nanosuspension based on steric stabilizers only [41]. 

### 3.2. TEM

**Figure 2:**
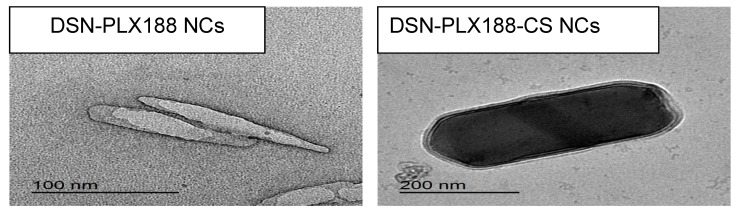
TEM microphotographs of the selected DSN NCs.

The morphology of the selected PLX188 and CS-PLX188 NCs examined using TEM is illustrated in Figure 2. DSN–PLX188 NCs appeared in the form of nanosized rods. TEM microphotographs showed the hydrodynamic layer of PLX188, which appeared as a grey layer surrounding NCs, reflecting the high affinity of the stabilizer towards the particle surface, providing steric stability. DSN–PLX188–CS NCs were larger nanometric rods surrounded by two layers of PLX188 and CS. It has been reported that cylindrical silica nanoparticles showed a higher accumulation in the liver than other shapes; thus, rod-shaped NCs can be expected to accumulate in the hepatic tissue and augment the therapeutic efficacy of the incorporated drugs [69]. 

TEM images revealed NCs with diameters smaller than that estimated by DLS. It has been reported that DLS measures the hydrodynamic diameter of the particles including the polymer(s) and the hydration layer; hence, larger particle diameters were recorded [70]. Additionally, the dispersion/aggregation behavior of NC dispersion greatly affects the size measurement by DLS [71]. 

### 3.3. SEM

**Figure 3:**
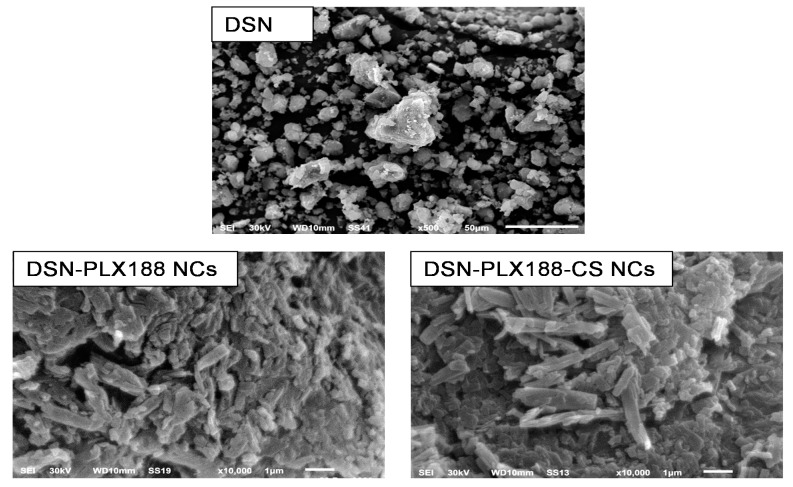
SEM microphotographs of DSN and the selected NCs.

The surface morphology of DSN powder and its selected NCs, employing PLX188 either alone or combined with CS, is presented in Figure 3. DSN showed microparticles with irregular shapes and a wide size distribution. In accordance with TEM examination, SEM images of DSN NCs based on either PLX188 or a PLX188–CS combination revealed nanometric rods with a smooth and uniform surface. Similar results have been reported elsewhere [45,72]. It has been found that sonoprecipitation could provide rod-shaped NCs that exhibited higher in vitro dissolution rates and bioavailability when compared to spherical ones with a similar hydrodynamic diameter [73]. Moreover, rod-shaped NCs can be expected to greatly accumulate in the liver and potentiate the therapeutic efficacy of the incorporated drugs [70]. Both SEM and TEM findings may indicate the benefits of our study over that previously adopted regarding DSN nanosuspension based on HPMC that incorporated oval-shaped crystals with one layer of a steric stabilizer adsorbed on their surfaces [41].

### 3.4. Solid-State Characterization 

#### 3.4.1. FT-IR

**Figure 4:**
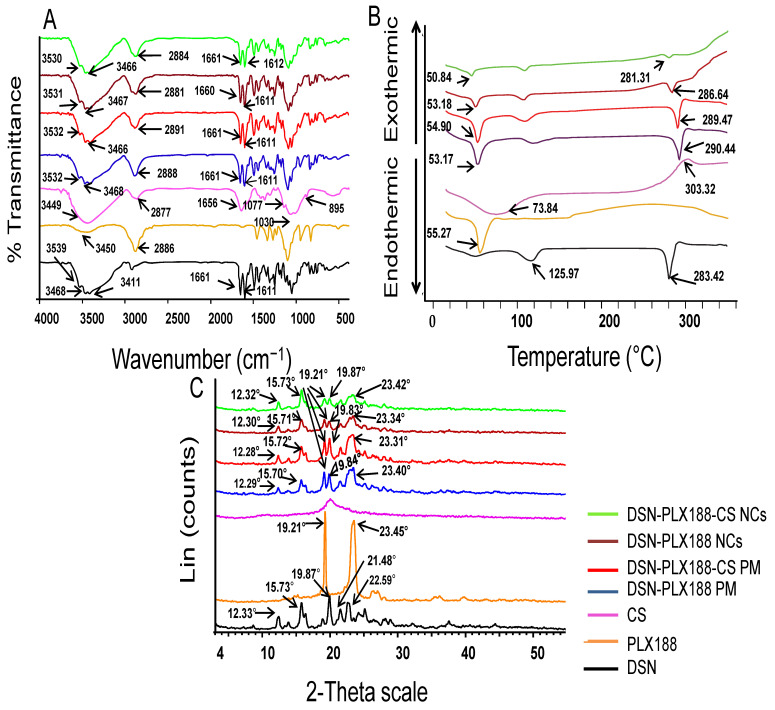
Solid-state characterization of the lyophilized selected NCs in comparison with DSN powder, individual polymers and the corresponding PMs. (**A**) FT-IR spectra, (**B**) DSC thermograms and (**C**) XRD patterns.

FT-IR spectroscopy was applied to assess the possibility of the interaction between DSN, PLX188, and CS. Figure 4A depicts the FT-IR spectra of DSN powder, PLX188, CS, and PMs of DSN and PLX188 with and without CS as well as the lyophilized PLX188 and PLX188–CS DSN NCs. The FT-IR spectrum of DSN exhibited characteristic bands at 3411, 3468, and 3539 cm^−1^ due to more than one O–H aromatic bond stretching as well as an absorption band at 1661 cm^−1^, corresponding to aromatic ketonic carbonyl stretching (C=O vibration) [26,41]. The stretching of (C=C) in the DSN aromatic ring resulted in two peaks at 1611 and 1502 cm^−1^ [26]. PLX188 had characteristic stretching vibration peaks at 3450 and 2886 cm^−1^, assigned to –OH and –CH3, respectively [74]. CS exhibited a characteristic strong and broad peak at 3449 cm^−1^ due to hydroxyl and amine group stretching vibrations. It also exhibited an absorption peak at 2877 cm^−1^ which may be attributed to C–H stretching. The peaks at 1656, 1030 and 1077 cm^−1^ corresponded to –C=O of the residual acetyl group, C–O and C–H groups, respectively. The band at 895 cm^−1^ was due to the β-(1,4) glucoside bond in CS [20]. The characteristic peaks of the drug and the polymer(s) were present in FT-IR spectra of the PMs of DSN and PLX188 either alone or together with CS; thus, no interaction between DSN and any of these polymers could be expected on physical mixing. FT-IR spectra of lyophilized DSN NCs based on either PLX188 or a PLX188–CS combination were similar to that of the corresponding PM. Hence, it can be suggested that the stabilizers were physically adsorbed on DSN NCs without a chemical interaction.

#### 3.4.2. DSC 

DSC thermograms of the previously mentioned samples under FT-IR are presented in Figure 4B. The DSC thermogram of DSN exhibits a sharp endothermic peak at 283.42 °C, indicating its melting point and crystalline nature, in addition to a broad endothermic peak at 125.97 °C due to its dehydration. Similar results have been reported elsewhere [37]. The DSC thermogram of PLX188 discloses its characteristic sharp endothermic peak at 55.27 °C [17]. The DSC thermogram of CS shows an initial endothermic peak at 73.84 °C, corresponding to its dehydration, and an exothermic peak at 303.32 °C, corresponding to its decomposition and depolymerization [47,75,76]. Regarding DSN PMs with PLX188 alone or combined with CS, the drug and the polymer(s) peaks were still observed, whereas the drug peak was broadened or its intensity was reduced in the thermograms of its NCs based on PLX188 alone or combined with CS. The melting enthalpy values of DSN as a raw drug, PLX188 NCs and PLX188–CS NCs were −181, −42 and −8.71 J/g, respectively. This could be a consequence of crystal size decreases [9], dilution by stabilizers [45] or drug crystallinity reductions. Accordingly, it can be said that DSC was not a discriminative tool for clarifying the effects on drug crystallinity. Thus, a further study employing XRD was required [45,49].

#### 3.4.3. XRD 

Figure 4C illustrates XRD patterns of the investigated samples stated under FT-IR and DSC. The DSN diffractogram shows several sharp diffraction peaks at 12.33°, 15.73°, 19.87°, 21.48°, 22.59° and 25.11°, reflecting its crystalline nature [37]. PLX188 exhibits characteristic peaks at 19.21° and 23.45° [17]. The amorphous nature of CS is reflected by the absence of sharp peaks in its diffractogram [75]. The drug characteristic peaks are still recorded in the diffractograms of its PMs with PLX188 and the PLX188–CS combination. The drug peak appeared in the diffractograms of the corresponding lyophilized NCs with reduced intensities. These results could be explained by crystal size decreases rather than amorphization [9]. The maintenance of the original crystalline state can be a merit for long-term stability [45]. These results may still highlight the benefits of our selected NCs over those reported that were based on HPMC and showed a partial amorphization that can negatively affect product stability [41]. 

### 3.5. Lyophilization of NC Dispersions

The size and PDI of the lyophilized powders of the selected DSN–PLX188 and DSN–PLX188–CS NCs with and without the addition of mannitol (1% *w*/*v*) were measured after reconstitution with double-deionized water and are listed in Table 3. Compared to both types of NC dispersions before lyophilization, there was an insignificant difference in the particle size and PDI values following their lyophilization with the addition of 1% *w*/*v* mannitol. On the other hand, those lyophilized without the addition of mannitol encountered a significant increase in the particle size and PDI when compared to the corresponding dispersions, either before or after lyophilization, employing mannitol as a cryoprotectant. These results confirmed that the addition of 1% *w*/*v* mannitol counteracted the particle aggregation of both types of NC dispersions on lyophilization. 

### 3.6. In Vitro Dissolution Study

**Figure 5:**
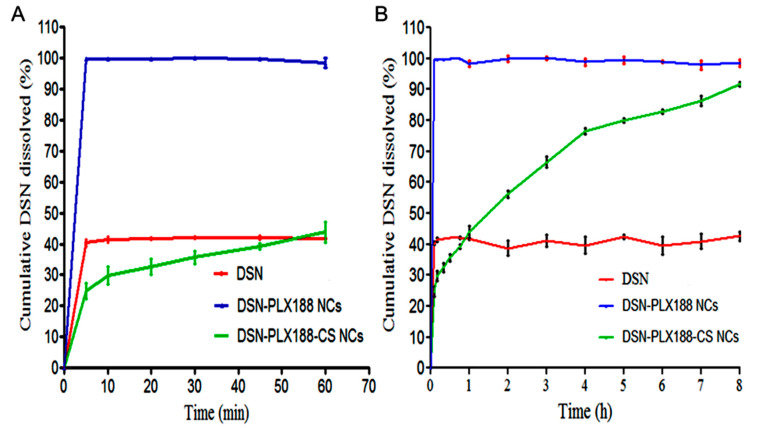
In vitro DSN dissolution up to (**A**) 60 min and (**B**) 8 h from the selected NCs based on PLX188 alone and the PLX188–CS combination in comparison with the drug in a sodium orthophosphate buffer (pH 12), employing the paddle method. Data are expressed as the mean ± SD (*n* = 3).

The dissolution behavior of the DSN crude drug was compared to that of its NCs based on PLX188 alone and CS-PLX188 combination at sodium orthophosphate buffer (pH 12), and the results are graphed up to 60 min (Figure 5A) and 8 h (Figure 5B). The dissolution parameters are illustrated in Table 4. In accordance with Figure 5, there was a remarkable enhancement of DSN dissolution from both types of NCs relative to the crude drug powder, as indicated by the significantly higher PD_2h_ and DE values of these NCs relative to the crude drug. The burst dissolution of DSN from its PLX188 NCs can be explained by the decrease in the interfacial tension and the expected increase in NC wettability due to PLX188 [1]. The poor wettability and the expected agglomeration of DSN may account for its low dissolution. In contrast to DSN–PLX188 NCs, there was a significant increase in MDT of DSN from its PLX188–CS NCs, reflecting the drug-controlled release, probably because of CS insolubility and its diffusion layer [49]. Generally, *f*_2_ was less than 50 for the studied NCs, indicating significantly different dissolution profiles of DSN from them when compared to that of the crude DSN powder [6,51]. The dissolution enhancement of DSN can be attributed to the size reduction to rod-shaped NCs that increased the available surface area, and hence, the drug wettability [49,77]. The controlled release of DSN due to CS may reveal another advantage of our selected NCs when compared to those reported that showed a burst release [41]. 

### 3.7. Kinetic Analysis of Release Data

Regarding DSN–PLX188–CS NCs, the highest coefficient of determination (R^2^ = 0.9973) was obtained in the case of the Higuchi model versus 0.9498 and 0.9877 for zero and first orders, respectively. Thus, in vitro release of DSN from its PLX188–CS NCs can be explained by the Higuchi model, suggesting a diffusion-controlled drug release. In accordance, the Fickian mechanism (*n* = 0.2953, R^2^ = 0.9825) was found to describe the drug release from these NCs, verifying the diffusion-controlled drug release [54]. Other drugs loaded in CS nanoparticles followed the same kinetic behavior [78].

### 3.8. Stability Studies

Table 5 presents the stability study results of the lyophilized PLX188 and PLX188–CS NCs of DSN over a period of three months at room temperature. There was a significant increase in the particle size of DSN–PLX188 NCs in the third month when compared to the particle size initially determined at the beginning of the study. The significantly lower potential of these NCs in the third month and the possible particle aggregation may explain their size enlargement in that month. On the other hand, there was no significant change in average DSN–PLX188–CS NC particle size, PDI, and zeta potential when compared to the initial respective measurements. These results may suggest the advantage of the combination of an electrostatic stabilizer (CS) and a steric stabilizer (PLX188) over the use of a steric stabilizer alone, such as what was adopted in a previous study of DSN nanosuspension [41]. The insignificant change in PDI values for both types of NCs reflected the maintenance of a uniform particle size distribution. Additionally, there was no significant change in the drug content for both types of NCs during the storage period.

### 3.9. In Vivo Evaluation

#### 3.9.1. Biomarker Assessment

The hepatocytes membrane damage can be indicated by the discharge of the intracellular enzymes including AST, ALT, ALP, GGT and LDH in blood circulation [34]. Elevated ALP levels can result from the increased biliary pressure that induces ALP synthesis [79]. Serum GGT has been used as a marker of liver dysfunction because the products of the GGT reaction may increase the free radical production in the case of iron overload [80]. It has been reported that excess iron can induce the gene expression of a liver enzyme called 3-hydroxy-3-methyl-3-glutaryl-coenzyme A (HMG-CoA) reductase, which participates in cholesterol synthesis, elevating serum levels of the dyslipidemia marker, TC [81]. ALB is the most essential protein synthesized in the liver and its serum concentration is a virtuous indicator of hepatic function integrity [82]. Increased serum TB levels indicate a serious liver injury because bilirubin is conjugated with glucuronic acid in the liver to then be excreted in the bile [82]. Iron overload greatly affects nitro oxidative stress markers because excess iron induces oxidative mitochondrial membrane injury and diminishes enzymes of the tri-carboxylic acid cycle [83]. The redox properties of iron can trigger the production of reactive oxygen species and hepatocyte destruction [84]. Additionally, iron plays an important role in the production of free radicals in the biological system [85]. Therefore, the hepatic tissue content of GSH and MDA, as well as NO_X_ production, can reflect the extent of hepatic injury.

**Figure 6:**
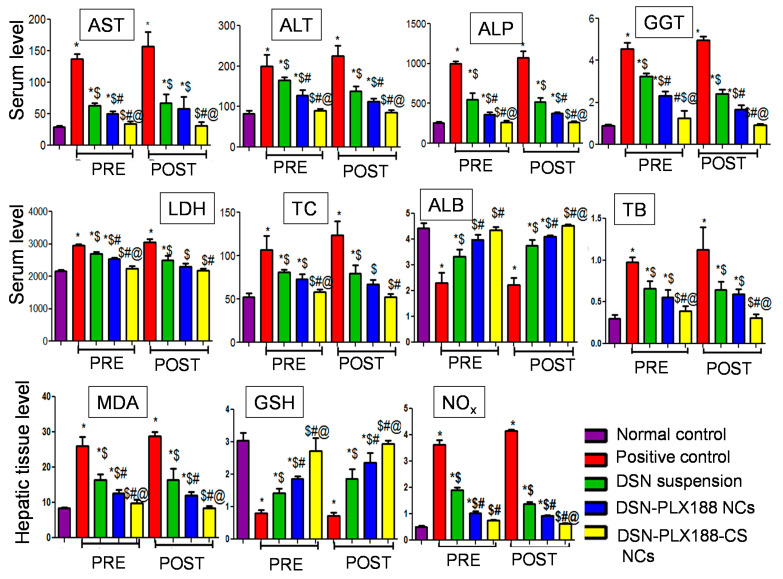
The serum/hepatic tissue levels of the examined biomarkers following the pretreatment (PRE) and the post-treatment (POST) regimens. Data are expressed as the mean ± SD (*n* = 6). The statistical analysis was performed at *p* < 0.05. *, $, # and @ indicate significant differences. * vs. the normal control. $ vs. the positive control. # vs. DSN-suspension-treated group. @ vs. DSN–PLX188-NC-treated group. AST, aspartate aminotransferase; ALT, alanine aminotransferase; ALP, alkaline phosphatase; GGT, gamma glutamyl transferase; LDH, lactate dehydrogenase; TC, total cholesterol; ALB, albumin; TB, total bilirubin; MDA, malondialdehyde; GSH, reduced glutathione; NO_x_, nitrate/nitrite production.

Figure 6 presents the serum and the tissue levels of the examined biomarkers in both pretreatment and post-treatment models. In both treatment regimens, the ability of ferrous sulfate to induce haptic injury in the positive control group was verified by the significant increase in the biomarker serum levels (except ALB) and the hepatic tissue contents of the oxidative stress (MDA) and the inflammatory (NO_X_) markers when compared to normal rats. Significantly reduced ALB serum levels and GSH tissue contents relative to normal rats were observed in the positive control rats. 

DSN was able to significantly diminish the levels of these markers (except ALB and GSH) relative to the positive control in both models, possibly due to its antioxidant and anti-inflammatory capabilities that maintain membrane permeability [86]. Moreover, TC serum levels were lowered by DSN, possibly due to the inhibition of HMG-CoA reductase and acyl CoA cholesterol acyltransferase (ACAT) [87]. However, the marker levels in the rats that received DSN suspension orally in both models were still higher than those determined for the normal control. Regarding ALB and GSH, DSN antioxidant capabilities seemed to impart elevated levels of these biomarkers relative to the positive control in both models, although their levels were still lower than those exhibited by the normal rats. 

Concerning PLX188 NCs of DSN, the serum and the tissue levels of the studied biomarkers (except ALB and GSH) were significantly lower than those recorded for the positive control and for most of the biomarkers relative to the drug suspension. The higher efficacy of DSN–PLX188 NCs over the drug against the liver damage can be explained by the increased drug solubility due to the nanometric rod-shaped NCs and the improved wettability, as well as P-glycoprotein inhibition and membrane interaction capabilities of PLX188 which could, together, enhance DSN bioavailability [18]. However, the biomarker levels were still significantly higher than those estimated for normal control rats except for LDH and TC in the post-treatment model. Although being significantly greater than the positive control rats, rats that administered PLX188 NCs of DSN showed significantly lower ALB and GSH levels when compared to normal rats.

The DSN–PLX188–CS-NC-administered group encountered significantly regulated levels of all or most of the studied biomarkers in both regimens when compared to those determined for the positive control as well as the rats that received the DSN suspension or PLX188 NCs. Moreover, there was an insignificant difference between the rats that were administered DSN–PLX188–CS NCs and normal controls regarding all biomarker levels. These results reflect the superiority of DSN–PLX188–CS NCs over the drug suspension and its PLX188-based NCs. The potentiation of DSN efficacy against liver injury by its formulation into PLX188–CS NCs was evident in the post-treatment model, as indicated by the significant reduction in NO_x_ expression in the hepatic tissue when compared to PLX188 NCs. In addition to the augmented drug solubility, CS mucoadhesive and permeability-enhancing properties, as well as the expected electrostatic interaction with the negatively charged cellular membranes, may account for the superiority of its NCs over that based on PLX188 alone and the drug suspension [22]. Additionally, post-treatment may have potentiated the uptake of the positively charged DSN–PLX188–CS NCs by the overexpressed Kupffer cells, which are abundant liver-resident macrophages [24]. Moreover, CS has been found to activate some antioxidant enzymes such as glutathione peroxidase [88,89].

#### 3.9.2. Histopathological Examination

**Figure 7:**
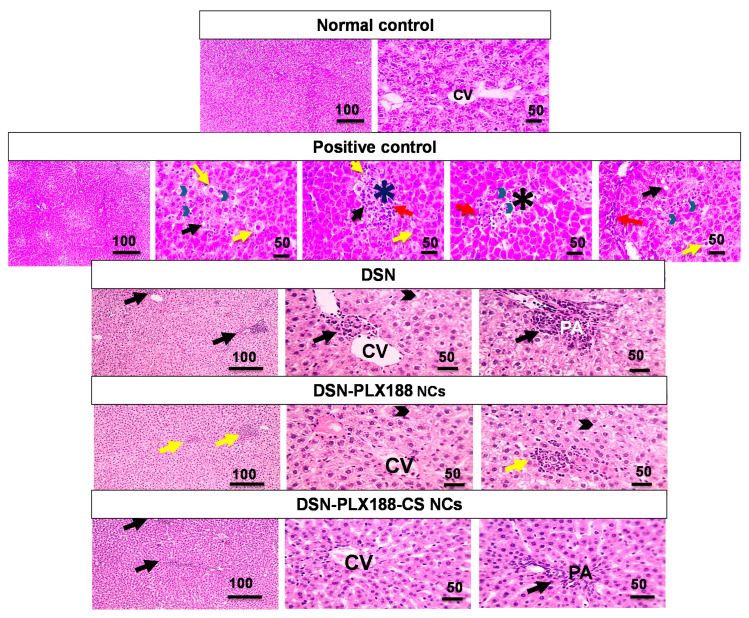
Microscopic pictures of hematoxylin and eosin (H&E)-stained hepatic sections of the different groups following the pretreatment regimen. Low magnification x: 100 bar 100 µm and high magnification Xx: 400 bar 50 µm. CV, central vein; PA, portal area. In the positive control: focal areas of necrosis (asterisks); apoptosis (arrowheads); vacuolization (black arrows); hydropic degeneration (yellow arrows); hepatocytes with infiltration of a few leukocytic cells (red arrows). In the DSN-pretreated group: leukocytic cell infiltration close to the central vein (black arrow); portal inflammation (black arrow); hydropic degeneration in hepatocytes (arrowheads). In the DSN–PLX188-NC-pretreated group: lobular inflammation (yellow arrows); hydropic degeneration (arrowheads). In the DSN–PLX188–CS-NC-pretreated group: leukocytic cell infiltration in portal area (black arrow).

Figure 7 displays the microphotographs of the histopathological examination of the liver tissue sections in the pretreatment model. Normal controls showed normal architecture of the hepatic tissue and arrangement of the hepatic cords around the central vein. Intraperitoneal injection of ferrous sulfate in the positive control resulted in a severe liver injury, as indicated by focal areas of necrosis, apoptosis, mild vacuolization, and hydropic degeneration of the hepatocytes with infiltration of a few leukocytic cells. Rats pretreated with the DSN suspension exhibited little leukocytic cell infiltration around the central vein, as well as mild portal inflammation and hydropic degeneration in the hepatocytes. Oral pretreatment with DSN–PLX188 NCs caused mild lobular inflammation and hydropic degeneration in the hepatocytes. 

**Figure 8:**
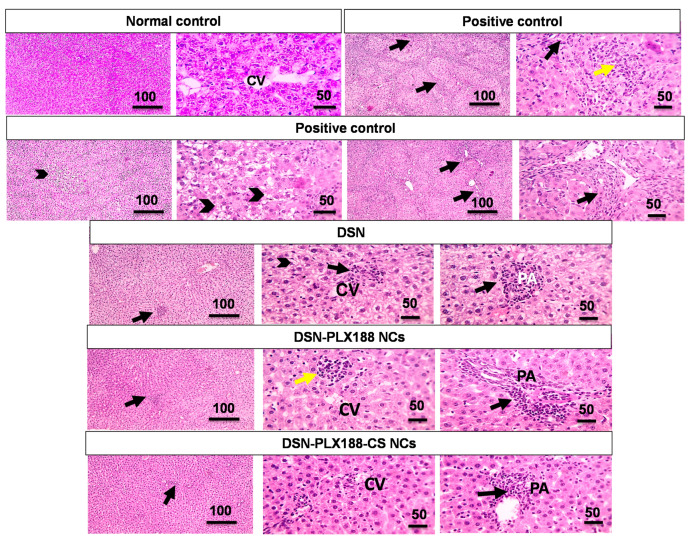
Microscopic pictures of hematoxylin and eosin (H&E)-stained hepatic sections of the different groups following the post-treatment regimen. Low magnification x: 100 bar 100 µm and high magnification x: 400 bar 50 µm. CV, central vein; PA, portal area. In the positive control: portal fibrosis and inflammation (black arrows); lobular inflammation (yellow arrow); hydropic degeneration in hepatocytes (arrowheads). In the DSN-post-treated group: lobular inflammation close to the central vein (black arrow); portal inflammation (black arrow); hydropic degeneration in the hepatocytes (arrowheads). In the DSN–PLX188-NC-post-treated group: lobular inflammation (yellow arrow); portal inflammation (black arrow). In the DSN–PLX188–CS-post-treated group: leukocytic cell infiltration in portal area (black arrow).

Figure 8 presents the microphotographs of the histopathological examination of the hepatic tissue sections in the post-treatment model. Similarly, hepatic damage in the positive control rats in the post-treatment model was recognized in the form of portal fibrosis and inflammation and a lobular inflammation with hydropic degeneration in the hepatocytes and marked leukocytic cell infiltration. Rats post-treated with DSN suspension showed mild lobular and portal inflammations as well as mild hydropic degeneration in the hepatocytes. In the post-treatment model, the rats that received PLX188 NCs orally experienced mild lobular and portal inflammations and normal arrangement and appearance of the hepatocytes.

Regarding DSN–PLX188–CS-NC-pretreated and post-treated groups (Figure 7 and Figure 8, respectively), infiltration of a few leukocytic cells in the portal area associated with normal arrangement and appearance of the hepatocytes was observed.

**Figure 9:**
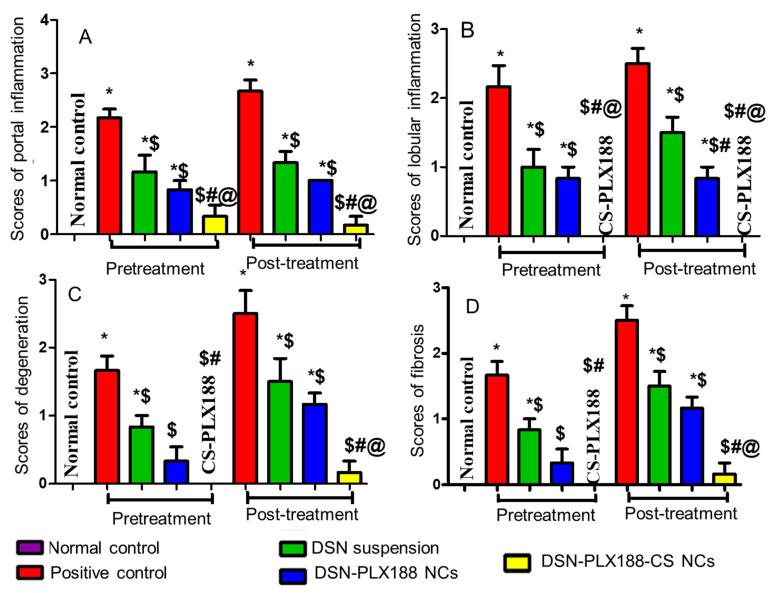
Statistical analysis of the histopathological alterations of the hepatic sections of the different groups following the pretreatment and the post-treatment regimens. (**A**) Portal inflammation, (**B**) Lobular inflammation, (**C**) Degeneration and (**D**) Fibrosis. Data are expressed as the mean ± SD. The statistical analysis was performed at *p* < 0.05. *, $, #, and @ indicate significant differences. * vs. the normal control. $ vs. the positive control. # vs. DSN-treated group. @ vs. DSN–PLX188-NC-treated group.

The statistical analysis results of the histopathological examination of the hepatic tissues in the pretreatment and the post-treatment models are depicted in Figure 9. The results indicated that there was a significant increase in the portal (A) and the lobular (B) inflammations, as well as the degeneration (C) and the fibrosis (D) in the positive control group when compared to the normal rats. Generally, oral pretreatment and post-treatment with the DSN suspension, DSN–PLX188 NCs and DSN–PLX188–CS NCs significantly lowered the pathologic scores and reduced the hepatic damage induced by ferrous sulfate relative to the positive control group. In comparison with DSN suspension, DSN–PLX188–CS NCs significantly attenuated all pathological changes in both treatment models. In both regimens, DSN–PLX188–CS-NC-treated groups exhibited a significant decrease in both portal (A) and lobular (B) inflammations in comparison with rats that orally received DSN NCs based on PLX188 alone. Regarding degeneration (C) and fibrosis (D), this superiority offered by NCs incorporating both polymers over PLX188 NCs was still observed in the post-treatment regimen only. These results agreed with the significant decline in the hepatic content of NO_x_ as indicative of hepatic tissue damage in the case of post-treatment only with DSN–PLX188–CS NCs when compared to DSN–PLX188 NCs (Figure 6). These results can still be explained on the basis that the post-treatment may have augmented the uptake of the positively charged DSN–PLX188–CS NCs by the overexpressed Kupffer cells [24]. 

In accordance with the biomarker assessment, the advantages of DSN–PLX188–CS NCs were verified by insignificant differences regarding all pathological alterations in comparison with normal rats. In addition to the solubility of the augmented drug, CS mucoadhesive and permeability-enhancing properties, as well as the expected increased uptake by Kupffer cells in the inflamed hepatic tissue, may still explain the superior performance of its NCs over that based on PLX188 alone and the drug suspension [22,24]. 

#### 3.9.3. Immunohistochemical Evaluation of Inducible Nitric Oxide Synthase (iNOS)

**Figure 10:**
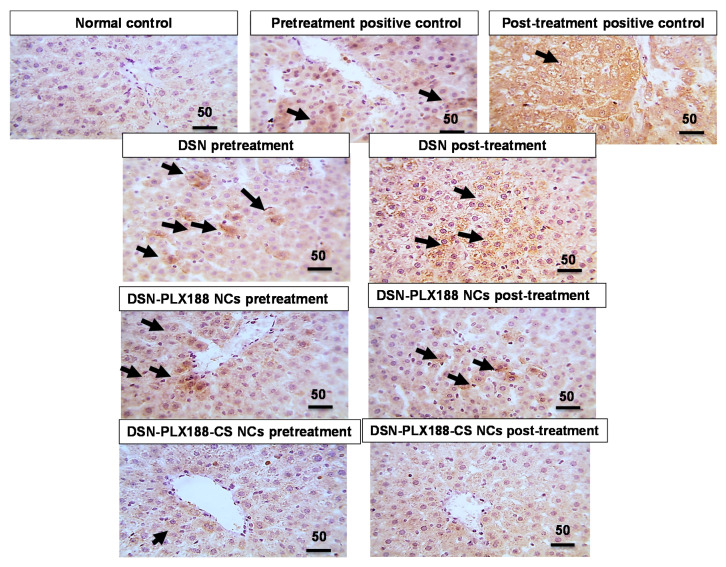
Microscopic pictures of immunostained hepatic sections against iNOS in the different groups following the pretreatment and the post-treatment regimens. High magnification x: 400 bar 50 µm. Positive expression against iNOS (black arrow).

Figure 10 illustrates the immunohistochemical assessment of iNOS expression in the hepatic tissue following both the pretreatment and the post-treatment models. Normal rats showed a negative expression against iNOS. The immunoreactivity against iNOS recorded in the hepatic sections of the positive control rats was either marked or moderate following the post-treatment and the pretreatment regimens, respectively. Accordingly, it has been reported that the activity of iNOS is up-regulated under pathological conditions, producing deleterious tissue damage due to NO_x_ overproduction and the subsequent oxido-nitrosative stress because of the interaction between nitrite and hydrogen peroxide that produces highly damaging peroxynitrite [90]. A mild positive expression against iNOS was recognized in the liver sections of rats that were pretreated with PLX188–CS NCs of DSN. The later immunoreactivity was increased slightly in rats pretreated with DSN–PLX188 NCs, and moderately in rats pretreated with the DSN suspension. Regarding the post-treatment regimen, a moderate positive hepatic expression against iNOS was recognized in rats that were orally administered the DSN suspension. There was mild positive iNOS activity recorded in the rats that were subsequently treated with DSN–PLX188 NCs. Negative iNOS expression was observed in the rats that were subsequently treated with DSN–PLX188–CS NCs. The decline in iNOS expression due to the pretreatment and the post-treatment with DSN as either suspension or NCs can mainly refer to its antioxidant and anti-inflammatory characteristics [91]. 

**Figure 11:**
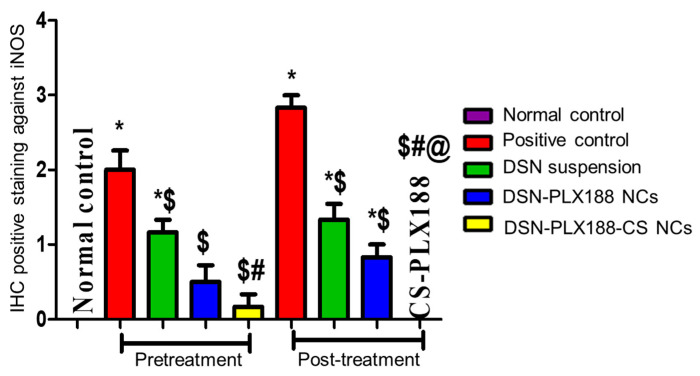
Statistical analysis of immunohistochemical (IHC) staining intensity of iNOS in the hepatic tissue of the different groups following the pretreatment and the post-treatment regimens. Data are presented as the mean ± SD. The statistical analysis was performed at *p* < 0.05. *, $, # and @ indicate significant differences. * vs. the normal control. $ vs. the positive control. # vs. DSN-treated group. @ vs. DSN–PLX188-NC-treated group.

The results of the statistical analysis of the immunohistochemical expression of iNOS were well-correlated with the above-mentioned results (Figure 11). This figure indicates a significant increase in hepatic iNOS expression in the positive control when compared to the normal rats. In general, the pretreatment and the post-treatment with DSN as either a suspension or NCs lowered the hepatic iNOS expression in comparison with the positive control. In accordance with both the biomarker assessment and histopathological examination, the superiority of PLX188–CS NCs was obvious, particularly in the post-treatment model by abolishing iNOS expression in the hepatic tissue to be insignificantly different from that recorded for the normal rats. The improved drug solubility by the size reduction to the nanometric rod-shaped NCs, in addition to CS mucoadhesive and permeability-enhancing properties, can still account for the superior performance of CS-PLX188 NCs [22]. In addition, the enhanced uptake by Kupffer cells in the inflamed hepatic tissue can also explain this superiority [24]. 

## 4. Conclusions

The optimized NCs were based on a combination of 0.15% *w*/*v* PLX188 as a steric stabilizer and 0.04% *w*/*v* CS as an electrostatic stabilizer. They appeared as rods according to SEM and TEM examinations. These NCs maintained their average size (368.93 ± 0.47 nm) and narrow size distribution (PDI = 0.23 ± 0.01) on lyophilization on the addition of mannitol (1% *w*/*v*). The stabilizers were physically adsorbed on DSN NC surfaces without a chemical interaction or drug amorphization, as revealed by the solid-state characterization. The nanometric rod-shaped NCs, the increased surface area, and the improved drug wettability can together account for the enhanced DSN dissolution from the selected NCs. DSN–PLX188–CS NCs were more stable than those based on PLX188 alone up to the third month at room temperature, suggesting the significance of the combined electrostatic and steric stabilization strategies. Assessment of therapeutic efficacy of the selected DSN–PLX188–CS NCs was performed; the results revealed their superiority, particularly in the post-treatment model, as indicated by the significantly regulated serum and tissue levels of most of the examined biomarkers as well as the significantly reduced pathological alterations and iNOS expression in the hepatic tissue in comparison with the positive control, DSN suspension and DSN–PLX188 NCs. The prevalence of DSN–PLX188–CS NCs was verified by the insignificant differences from normal rats regarding the above-mentioned parameters. Therefore, DSN–PLX188–CS NCs can be defined as a promising drug delivery system for improving DSN dissolution and therapeutic efficacy against ferrous-sulfate-induced hepatic injury in rats. 

## Figures and Tables

**Table 1:** Particle size analysis of the prepared NC dispersions.

Stabilizer	Concentration (% *w*/*v*)	Size (nm)	PDI
PVP K25	0.1	578.53 ± 16.98	0.32 ± 0.06
	0.15	401.20 ± 16.48 *	0.25 ± 0.02
	0.3	466.47 ± 11.34 *^#^	0.30 ± 0.03
	0.6	662.57 ± 22.70 *^#@^	0.55 ± 0.02
PLX188	0.1	401.66 ± 54.50	0.42 ± 0.11
	0.15	350.20 ± 1.79 *	0.24 ± 0.01
	0.3	353.30 ± 2.50 *	0.22 ± 0.01
	0.6	528.03 ± 3.53 *^#@^	0.36 ± 0.03
SDS	0.1	968.03 ± 66.70	0.49 ± 0.05
	0.15	828.27 ± 54.36 *	0.42 ± 0.03
	0.3	527.33 ± 10.01 *^#^	0.40 ± 0.05
	0.6	832.09 ± 21.87 *^@^	0.22 ± 0.05
CS/PLX188	0.02/0.15	443.13 ± 6.05 ^$^	0.35 ± 0.02
	0.04/0.15	368.93 ± 0.47 *	0.23 ± 0.01
	0.06/0.15	453.17± 11.71 ^#$^	0.25 ± 0.04

Data are presented as the mean ± SD (*n* = 3). The statistical analysis was performed at *p* < 0.05. *, ^#^, ^$^, and ^@^ indicate significant differences. Regarding PVP K25, SDS, and PLX188: * vs. 0.1% *w*/*v*; ^#^ vs. 0.15% *w*/*v*; ^@^ vs. 0.3% *w*/*v*. Regarding CS/PLX188 combinations: * vs. 0.02/0.15 (% *w*/*v*); ^#^ vs. 0.04%/0.15 (% *w*/*v*); ^$^ vs. 0.15% *w*/*v* PLX188 alone. PVP K25, poly vinyl pyrrolidone K25; PLX188, poloxamer 188; SDS, sodium dodecyl sulfate; CS, chitosan; PDI, poly dispersity index.

**Table 2:** ζ-potential of the selected NC dispersions.

Stabilizer (% *w*/*v*)	ζ-Potential (mV)
0.1 PLX188	−19.91 ± 0.23
0.15 PLX188	−25.93 ± 0.30 *
0.3% PLX188	−21.33 ± 0.36 ^#^
0.6% PLX188	−18.67 ± 0.40 ^#@^
0.02% CS/0.15% PLX188	+25.90 ± 0.44
0.04% CS/0.15% PLX188	+40.43 ± 0.15 *
0.06% CS/0.15% PLX188	+43.50 ± 0.78 *

Data are expressed as the mean ± SD (*n* = 3). The statistical analysis was performed at *p* < 0.05. *, ^#^, and ^@^ indicate significant differences. Regarding PLX188 NCs: * vs. 0.1% *w*/*v*; ^#^ vs. 0.15% *w*/*v*; ^@^ vs. 0.3% *w*/*v*. Regarding CS/PLX188 combinations: * vs. 0.02/0.15 (% *w*/*v*); ^#^ vs. 0.04/0.15 (% *w*/*v*).

**Table 3:** Lyophilization effects on the size and the size distribution of the selected NCs.

Formula	Size (nm)	PDI
DSN–PLX188 NCs		
Before lyophilization	350.20 ± 1.79	0.24 ± 0.01
After lyophilization with 1% *w*/*v* mannitol	349.43 ± 5.75	0.24 ± 0.02
After lyophilization without 1% *w*/*v* mannitol	487.53 ± 26.65 *^#^	0.54 ± 0.02 *^#^
DSN–PLX188–CS NCs		
Before lyophilization	368.93 ± 0.47	0.21 ± 0.03
After lyophilization with 1% *w*/*v* mannitol	360.36 ± 7.07	0.24 ± 0.03
After lyophilization without 1% *w*/*v* mannitol	408.50 ± 12.27 *^#^	0.386 ± 0.03 *^#^

Data are presented as the mean ± SD. The statistical analysis was performed at *p* < 0.05. *, and ^#^ indicate significant differences. * vs. formula before lyophilization; ^#^ vs. formula after lyophilization with 1% *w*/*v* mannitol.

**Table 4:** The dissolution parameters of the selected NCs in comparison with DSN.

Parameter	DSN	DSN–PLX188 NCs	DSN–PLX188–CS NCs
DE	40.59 ± 2.68	98.67 ± 1.30 *	68.79 ± 1.05 *^$^
MDT	0.38 ± 0.26	0.03 ± 0.02 *	2.00 ± 0.06 *^$^
PD_2h_	38.70 ± 4.24	99.94 ± 1.46 *	56.18 ± 1.93 *^$^
*f*_2_	NA	8.90 ± 0.88	24.31 ± 1.14

Data are expressed as the mean ± SD. The statistical analysis was performed at *p* < 0.05. * and ^$^ indicate significant differences. * vs. DSN; ^$^ vs. DSN–PLX188 NCs. DE, dissolution efficiency; MDT, mean dissolution time; PD_2h_, percent dissolved after 2 h; *f*_2_, similarity factor and NA, non-applicable.

**Table 5:** Storage stability of the selected lyophilized NCs at room temperature.

Month	Size (nm)	PDI	Drug Content (%)	ζ-Potential (mV)
DSN–PLX188 NCs				
0	349.43 ± 5.75	0.24 ± 0.02	93.12 ± 0.57	−25.17 ± 0.38
1	349.83 ± 5.22	0.21 ± 0.03	93.32 ± 0.30	−24.80 ± 0.30
2	362.60 ± 7.74	0.25 ± 0.02	93.20 ± 0.55	−23.63 ± 0.65
3	374.87 ± 3.11 *	0.19 ± 0.01	92.83 ± 0.83	−21.86 ± 0.75 *
DSN–PLX188–CS NCs				
0	360.63 ± 7.07	0.21 ± 0.03	93.29 ± 0.58	40.43 ± 0.15
1	358.97 ± 6.27	0.22 ± 0.02	92.91 ± 0.51	39.63 ± 1.42
2	364.80 ± 4.49	0.25 ± 0.02	93.08 ± 0.74	39.17 ± 0.81
3	355.83 ± 7.86	0.19 ± 0.01	93.03 ± 1.21	39.60 ± 0.36

Data are presented as the mean ± SD. The statistical analysis was performed at *p* < 0.05. * indicates a significant difference vs. the corresponding initial measurements at zero time.

## Data Availability

Not applicable.
